# Primary transcriptome map of the hyperthermophilic archaeon *Thermococcus kodakarensis*

**DOI:** 10.1186/1471-2164-15-684

**Published:** 2014-08-16

**Authors:** Dominik Jäger, Konrad U Förstner, Cynthia M Sharma, Thomas J Santangelo, John N Reeve

**Affiliations:** Department of Microbiology, Ohio State University, 484 West 12th Ave, Columbus, OH 43210 USA; Research Center for Infectious Diseases, University of Würzburg, Würzburg, Germany; Institute for Molecular Infection Biology, University of Würzburg, Würzburg, Germany; Department of Biochemistry and Molecular Biology, Colorado State University, Fort Collins, CO 80523 USA

**Keywords:** Transcriptome, *Archaea*, Promoters, Antisense RNAs, Small non-coding RNAs, Riboswitch, Hyperthermophile, Hydrogen regulation

## Abstract

**Background:**

Prokaryotes have relatively small genomes, densely-packed with protein-encoding sequences. RNA sequencing has, however, revealed surprisingly complex transcriptomes and here we report the transcripts present in the model hyperthermophilic *Archaeon*, *Thermococcus kodakarensis*, under different physiological conditions.

**Results:**

Sequencing cDNA libraries, generated from RNA isolated from cells under different growth and metabolic conditions has identified >2,700 sites of transcription initiation, established a genome-wide map of transcripts*,* and consensus sequences for transcription initiation and post-transcription regulatory elements. The primary transcription start sites (TSS) upstream of 1,254 annotated genes, plus 644 primary TSS and their promoters within genes, are identified. Most mRNAs have a 5'-untranslated region (5'-UTR) 10 to 50 nt long (median = 16 nt), but ~20% have 5'-UTRs from 50 to 300 nt long and ~14% are leaderless. Approximately 50% of mRNAs contain a consensus ribosome binding sequence. The results identify TSS for 1,018 antisense transcripts, most with sequences complementary to either the 5'- or 3'-region of a sense mRNA, and confirm the presence of transcripts from all three CRISPR loci, the RNase P and 7S RNAs, all tRNAs and rRNAs and 69 predicted snoRNAs. Two putative riboswitch RNAs were present in growing but not in stationary phase cells. The procedure used is designed to identify TSS but, assuming that the number of cDNA reads correlates with transcript abundance, the results also provide a semi-quantitative documentation of the differences in *T. kodakarensis* genome expression under different growth conditions and confirm previous observations of substrate-dependent specific gene expression. Many previously unanticipated small RNAs have been identified, some with relative low GC contents (≤50%) and sequences that do not fold readily into base-paired secondary structures, contrary to the classical expectations for non-coding RNAs in a hyperthermophile.

**Conclusion:**

The results identify >2,700 TSS, including almost all of the primary sites of transcription initiation upstream of annotated genes, plus many secondary sites, sites within genes and sites resulting in antisense transcripts. The *T. kodakarensis* genome is small (~2.1 Mbp) and tightly packed with protein-encoding genes, but the transcriptomes established also contain many non-coding RNAs and predict extensive RNA-based regulation in this model *Archaeon*.

**Electronic supplementary material:**

The online version of this article (doi:10.1186/1471-2164-15-684) contains supplementary material, which is available to authorized users.

## Background

*Archaea* are prokaryotes, they resemble *Bacteria* in genome size, genome organization and the absence of a nuclear membrane, but their genetic information storage and expression components are generally more closely related to their eukaryotic than bacterial counterparts [1]. Historically, difficulties in manipulating *Archaea* genetically limited archaeal research but, with the discovery that *Thermococcus kodakarensis* is naturally competent for DNA uptake and transformation [2], genetic tools have been developed and *T. kodakarensis* established as a readily tractable experimental model for archaeal and hyperthermophile research [3]. As a fermentative heterotroph that grows rapidly, optimally at 85°C on a range of different substrates, *T. kodakarensis* offers opportunities to investigate archaeal gene regulation and metabolism under a variety of growth conditions and, as a hydrogen-producer, it has also attracted biotechnology attention. By using high-throughput RNA sequencing (RNA-seq), it is now possible to identify essentially all transcripts present in cells [4, 5] and such studies have revealed surprisingly complex transcriptomes in *Bacteria,* with many previously unanticipated non-coding small (sRNA) and antisense RNAs [6–20]. To add to this database, and specifically to add to the relatively few RNA-seq studies reported to date for *Archaea*[21–31], we have used differential RNA-seq technology (dRNA-seq) to identify the transcripts present in *T. kodakarensis* cells growing on different substrates and in stationary-phase cells. An automated analysis [15] was used to identify the sites at which the transcripts were initiated (transcription start sites; TSS) throughout the genome. Based on the conservation of sequences upstream of the TSS identified, consensus sequences have been identified for the core elements of *T. kodakarensis* promoters from which the synthesis of primary, secondary, internal and antisense transcripts is initiated.

## Results

### *T. kodakarensis*transcripts and transcription start sites (TSS)

**Figure 1:**
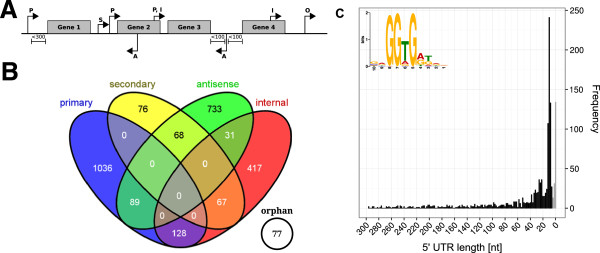
**Transcription start site (TSS) classification. (A)** Diagram showing all potential locations of TSS. In this report, they are categorized as primary (pTSS; most abundant transcript based on the highest number of cDNA reads) or secondary TSS (sTSS; all other transcripts) when located ≤300 bp upstream of a gene with transcription on the sense strand of that gene. They are categorized as internal TSS (iTSS) when located within an annotated gene and antisense TSS (aTSS) when located either in, or within ≤100 bp of a gene, on the antisense strand. A TSS not readily assigned to any of these categories was categorized as an orphan TSS (oTSS). **(B)** The distribution and overlap of the TSSs identified in each category. **(C)** The distribution of lengths of 5′-UTRs based on the combined number of pTSS and sTSS that initiate transcription at each position, with 0 being the first bp of the translation start codon. The numbers of TSS resulting in 5*'*-UTRs ≤8 nt, and so considered leaderless mRNAs, are indicated by the grey bars. The insert shows the consensus of the RBS identified in *T. kodakarensis*.

Identifying transcripts and precisely mapping transcription start sites (TSS) refines and extends genome annotation and the discovery of regulatory elements that control gene expression. To obtain this knowledge for *T. kodakarensis*, we employed differential high-throughput sequencing of cDNA libraries (dRNA-seq) generated from RNA preparations isolated from cells grown with and without sulfur, growth conditions that result in different patterns of gene expression and metabolism [32–35]. Specifically, cDNA libraries were generated and sequenced from RNA isolated from *T. kodakarensis* cells growing exponentially (S_exp_) and to stationary phase (S_stat_) in ASW-YT medium with sulfur, growing exponentially in ASW-YT with pyruvate (P_exp_), and from cells growing exponentially in pyruvate but 20 min after sulfur addition (PS). The cDNAs were generated after first incubating the RNA preparations with terminator exonuclease (TEX). TEX does not degrade primary transcripts with a 5′-triphosphate [7] but does digest RNAs generated by transcript processing that have a 5′-monophosphate. As a control and to fully document all transcripts, a cDNA library (C) was also generated and sequenced from an aliquot of an RNA preparation isolated from the cells growing exponentially with sulfur that was not exposed to TEX digestion. A total of ~32 million cDNA sequencing reads were obtained with 5.7 to 8.1 million reads generated from each individual library (Additional file 1: Table S1). After trimming, removal of poly(A)-tailed sequences and sequences shorter than 12 nt, 89% to 98% of the remaining sequences in each library mapped unequivocally to the *T. kodakarensis* genome [15, 36, 37]. cDNA sequences generated from dRNA-seq libraries cluster at the +1 site of the transcript [7] and this enrichment helps identify TSS and their associated upstream regulatory motifs. The sequences obtained were first evaluated, using an automated TSS identification method [15] that was provided with the *T. kodakarensis* genome annotation extended with known and predicted RNAs from the RFAM database [38] and from the UCSC archaeal genome browser [39]. A total of 2,718 TSS were identified and categorized based on their locations relative to annotated genes (Figure 1A). Primary and secondary TSS were defined as those initiating transcription on the sense strand and located upstream and within 300 bp of an annotated gene. The primary TSS (pTSS) was defined as that for which the most sequencing reads were obtained, and all other TSS assigned to the same transcriptional unit were designated secondary TSS (sTSS). TSS located within an annotated gene were designated internal TSS (iTSS) and when the TSS was located on the antisense strand in, or within 100 bp of an annotated gene, it was designated an antisense TSS (aTSS). As illustrated (Figures 1A and 1B), in some cases, an iTSS was potentially the pTSS or sTSS for a downstream gene and some aTSS were potentially the pTSS or sTSS for a gene on the complementary DNA strand. In these cases, the TSS was included in both categories, generating four categories that in total contained 3,204 TSS (Additional file 2: Table S2). There are 2,306 ORFs annotated in the *T. kodakarensis* genome [37] and 1,523 of these are predicted, by the DOOR^2^ database, to be organized into 507 multi-gene operons [40, 41]. A total of 1,254 pTSS were identified, and these include a pTSS identified upstream of ~78% of the predicted protein-encoding transcription units. Six TSS have been reported from experimental studies of *T. kodakarensis*[42–44], five match the pTSS (within ±1 nucleotide) identified here for that transcriptional unit (Additional file 3: Table S3). In the sixth case (TK0669, encodes cdc48), the pTSS identified is 679 bp upstream of the experimentally determined TSS [42]. Based on an overlapping ATGA-arrangement of translation initiation and termination codons, TK0670-TK0669 very likely form an operon with TK0669 being the promoter-distal gene. The location of the TSS identified here is consistent with the pTSS for a transcript of this operon.

### Promoters

**Figure 2:**
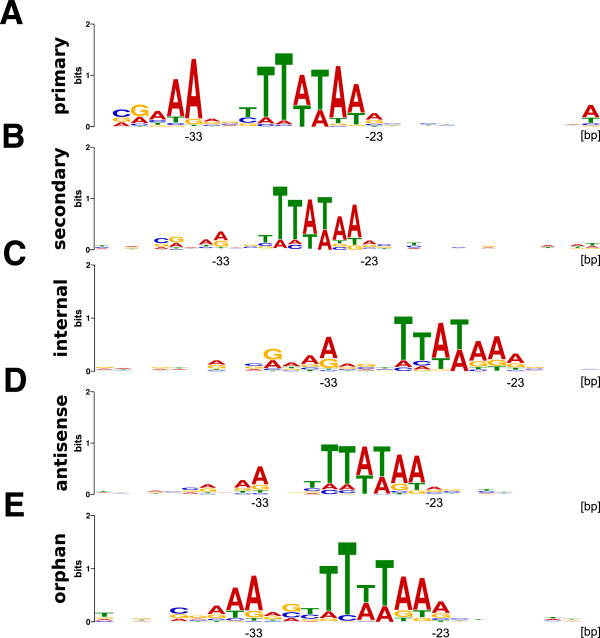
**Consensus promoter motifs.** Each frame shows the best ranking sequence motif identified by a *MEME* search [45] performed on a window from -50 bp upstream to the TSS for **(A)** pTSS of annotated ORFs, **(B)** sTSS of annotated ORFs, **(C)** iTSS and **(D)** aTSS. An orphan transcript was so designated when there was no detectable association of a TSS with an annotated gene, and **(E)** shows the consensus promoter motifs for orphan transcripts. The negative numbers on the x-axis indicate the distance in bp upstream from the identified TSS.

Archaeal promoters typically have a B recognition element (BRE) followed by a TATA-box and direct transcription initiation ~23 bp downstream from the TATA-box sequence [45]. To screen for promoter motifs, the sequences from -50 bp upstream to all identified TSS were analyzed using *MEME*[46]. This confirmed the presence of a TATA-box motif, located at a median distance of 23 bp, upstream of 96% and 88% of all the pTSS and sTSS, respectively (Figures 2A and 2B). A BRE motif, positioned upstream of the TATA-box, at a median distance of 33 bp from the TSS, was also clearly conserved although there was more sequence variability in the BREs upstream of sTSS presumably correlating with the sTSS being so designated based on lower expression. It is also possible that some TSS, designated sTSS, are not functionally associated with the downstream ORF but rather are the TSS of orphan transcripts directed by promoters that have more divergent sequences. BRE and TATA-box motifs are also present upstream of ~95% of the iTSS, some of which are likely to be the pTSS for downstream genes (Figure 2C). Consensus BRE and TATA-box motifs are also present upstream of most TSS that result in the synthesis of orphan small non-coding transcripts (sRNA) and antisense transcripts (asRNA) (Figures 2D and 2E), arguing that these are discrete gene products rather than products of random transcription or RNA processing as concluded for the majority of sRNAs and asRNAs in *Pyrococcus abyssi*[31].

### 5′-untranslated regions (5′-UTRs) and leaderless mRNAs

Archaeal mRNAs typically have short 5′-UTRs and, in some species, many 5′-UTRs are ≤8 nt and, as such, are designated as leaderless and are translated using a distinct initiation mechanism [47–49]. Based on the TSS identified, the majority of the mRNAs in *T. kodakarensis* have a 5′-UTR between 10 and 50 nt in length, with the median length being 16 nt. A ribosome binding sequence (RBS) that conforms to the consensus GGDGRD is present in ~50% of the predicted mRNAs (Figure 1C). Initially, we identified 179 leaderless mRNAs based on having a 5′-UTR ≤8 nt long, most of which encode proteins with unknown functions although 15 have annotated functions related to RNA processing and modification. To confirm that these were leaderless transcripts, a 100 bp window around each TSS was checked for the presence of a RBS using *FIMO*[46] and for alternative translation initiating codons. In 28 cases, this revealed evidence against the leaderless mRNA designation (Additional file 4: Table S4). Most often, a GTG codon was annotated as the translation initiating codon but an ATG codon was also present, in-frame, located 2 or 3 codons downstream within the ORF. With this ATG codon designated as the start codon, the 5′-UTR was extended and the transcript no longer conformed to the definition of a leaderless mRNA.

### Transcripts with long 5′-untranslated regions (5′-UTRs)

**Figure 3:**
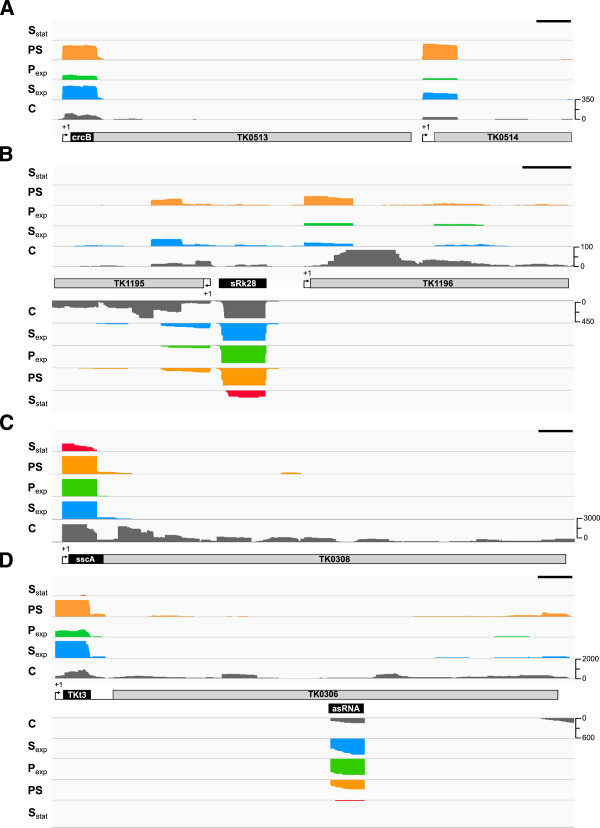
**Location and expression of potential regulatory RNAs.** Transcript abundances, based on cDNA reads, from regions proposed to function as **(A)** a fluoride-sensing riboswitch upstream of TK0513 [51] and **(B)** a pre-Q1 riboswitch designated sRk28. **(C)** Transcripts of sscA [24, 57] present in the 5*'*-UTR of TK0308. **(D)** Transcripts of tRNA^Lys^ (Tkt03) present within the 5*'*-UTR of TK0306. An antisense RNA is also transcribed from the TK0306 region. The numbers of cDNA reads from transcripts present in *T. kodakarensis* cells growing exponentially with sulfur (S_exp_; blue) and in stationary phase in sulfur medium (S_stat_; red), growing exponentially in pyruvate medium before (P_exp_; green) and 20 min after sulfur addition (PS; orange) are given by the peak heights. Data from the control library (C; exponential phase with sulfur) not digested with TEX are shown in grey. The relative abundance scales on the right of each panel allow direct comparisons of all data in that panel. The black scale bar in the top right corner of each panel is corresponds to 100 nt.

In total, 245 mRNAs were identified with long 5′-UTRs, defined as between 50 and 300 nt in length, with a median length of 103 nt. These 5′-UTRs have sequences ranging from 24% to 66% GC, with a mean of 48% GC, only slightly higher than the average 42% GC content of protein-encoding sequences, arguing against secondary structures stabilized by extensive G:C pairing. Most of these mRNAs encode hypothetical proteins, but 20 encode transporters, 11 encode aminoacyl-tRNA biosynthesis functions and 21 encode ribosomal proteins (21), data consistent with results from *P. abyssi* and *Sulfolobus solfataricus*[22, 32]. Transcripts with long 5′-UTRs were also identified in *Methanosarcina mazei*, suggesting post-transcription regulatory role [21] but, to date, there is only very limited experimental evidence for archaeal 5′-UTRs having regulatory functions [47, 50]. There is strong *in silico* support for riboswitches [51, 52] and our results confirm the presence of transcripts predicted to function as fluoride-sensing [51] and pre-Q1 sensing [23] riboswitches in the *Thermococcales*. The 86 nt putative fluoride-sensing riboswitch, designated the crcB RNA, is present in growing cells, but was not detected in RNA from stationary phase cells (Figure 3A) and, surprisingly, is encoded upstream of TK0513 rather than TK0514 (*crcB*), the gene predicted to encode a fluoride exporter [53, 54]. It remains possible that TK0513-TK0514 are cotranscribed, under some circumstances, but the pTSS for TK0514 is located between TK0513 and TK0514 arguing that TK0514 expression is not predominantly subject to crcB RNA regulation (Figure 3A). The presence of the crcB RNA in *P. abyssi* was also confirmed by RNA-seq [31]. Synthesis of the putative pre-Q1 sensing riboswitch [23], designated sRk28 in *P. abyssi,* is also growth phase regulated in *T. kodakarensis* but this RNA appears to be a small orphan transcript in *T. kodakarensis* (Figure 3B) rather than present within the 5′-UTR of a mRNA as in *P. abyssi*[31]. Although a riboswitch function has not been proven, there is evidence for riboswitch sequences interacting *in trans* with potential target mRNAs and thereby modulating gene expression [54, 55]. The two sRk28 RNAs do have very similar sequences, but they are encoded in different genome contexts. Downstream of the sRk28 encoding DNA in *T. kodakarensis* (Additional file 5: Figure S1) is a BRE-TATA box sequence and a TSS arguing that the immediately downstream gene (TK1195) is independently transcribed (Figure 3B).

Klein *et al.*[56] predicted the presence of a regulatory RNA, designated the sscA RNA, located upstream of the gene (TK0308) that encodes the translation elongation factor 1α (aEF1α). Our results confirm that the sscA RNA is present and abundant in growing *T. kodakarensis* cells and that it is located within the 118 nt 5′-UTR of the TK0308 mRNA (Figure 3C). The function of the sscA RNA remains to be determined, but its abundance increases with sulfur addition, and its location suggests a role in regulating translation. The transcript of a nearby gene (TK0306), that encodes a DEAD-box RNA helicase, was also reported to have a long 158 nt 5′-UTR [44]. Our results confirm the presence of this long 5′-UTR, demonstrate that it is actually 159 nt and reveal that it contains a tRNA^Lys^ (TKt3) apparently therefore co-transcribed with TK0306 (Figure 3D). A short (~70 nt) antisense RNA is also transcribed from within the TK0306 that is less abundant in cells growing with sulfur.

### Internal transcription start sites (iTSS)

**Figure 4:**
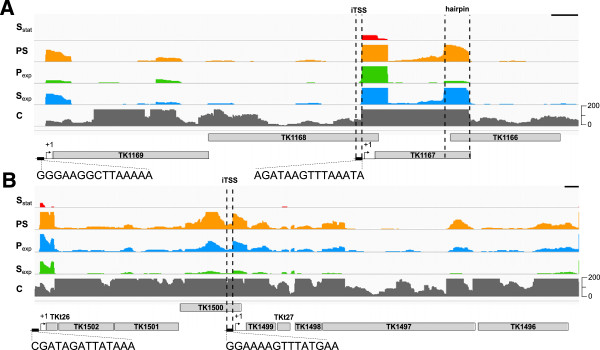
**Internal transcription start sites.** The genome organizations surrounding **(A)** rpoL (TK1169) and **(B)** rpoN (TK1499). The promoter motifs for the pTSS of TK1169 and TKt26 and for the iTSS identified for rpoL and rpoN are shown below the panels. The abundances of transcripts present in *T. kodakarensis* cells growing exponentially (S_exp_; blue) and in stationary phase (S_stat_; red) in sulfur medium, growing exponentially in pyruvate medium before (P_exp_; green) and 20 min after sulfur addition (PS; orange) are given by the peak heights. Data from the control library (C) not digested with TEX are shown in grey. The position of the sequence near the 3′-terminus of rpoL that, when transcribed, is predicted to form a stable RNA hairpin structure is indicated (see also Additional file 7: Figure S3). The black scale bar in the top right corner of each panel is corresponds to 100 nt.

In total, 644 TSS were identified within protein-encoding sequences and so are designated iTSS. Of these, 194 (~30%) are close to the 3′-terminus of an ORF and 125 (~20%) were automatically assigned by the analysis software as the pTSS of a downstream gene. A detailed individual review confirmed that 90 were most likely the pTSS of a downstream gene with BRE-TATA box motifs located appropriately upstream within the ORF. As illustrated (Figure 4), these include the TSS for TK1167 and TK1501, the genes that encode the rpoL and rpoN subunits of the *T. kodakarensis* RNA polymerase, respectively. There is also a region near the 3′-terminus of the rpoL transcript that resists TEX digestion, consistent with the sequence folding into a stable base-paired secondary structure. This is not a feature unique to the rpoL transcript. Additional file 6: Figure S2 shows several more examples of 3′-terminal regions of *T. kodakarensis* transcripts that resist TEX digestion and could fold into stable base-paired secondary structures.

**Figure 5:**
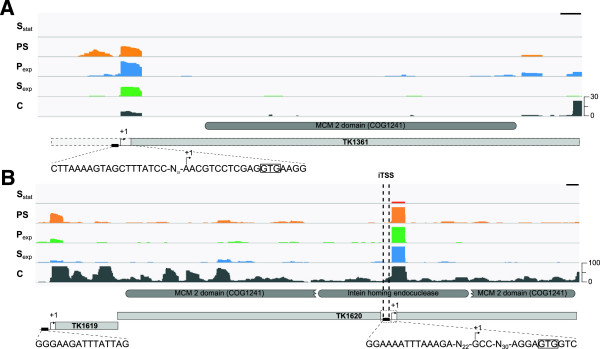
**Transcription of TK1361 (MCM2) and TK1620 (MCM3). (A)** As annotated in the *T. kodakarensis* genome [38], TK1361 has an atypical 5*'*-extension, here shown by broken lines. The location of the TSS identified for TK1361 and the sequence downstream that could function as a translation start site are shown. With the TSS re-categorized from iTSS to pTSS and translation initiated at the boxed GTG codon, the MCM2 generated has a standard MCM structure. **(B)** The organization of the TK1619-TK1620 (MCM3) region. The locations and upstream promoter sequences for the pTSS and an iTSS within TK1620 are indicated. A putative GTG translation initiation codon downstream of the iTSS is boxed. In both panels the protein domains, identified in the NCBI Conserved Domain Database [58], are shown in dark grey. The abundances of transcripts present in *T. kodakarensis* cells growing exponentially (S_exp_; blue) and in stationary phase (S_stat_; red) in sulfur medium, growing exponentially in pyruvate medium before (P_exp_; green) and 20 min after sulfur addition (PS; orange) are given by the peak heights. Data from the control library (C) not digested with TEX are shown in grey. The relative abundance scales on the right of each panel allow direct comparisons of all data in that panel. The black scale bar in the top right corner of each panel is corresponds to 100 nt.

Initially, 51 iTSS were identified near the 5′-termini of ORFs but, in 35 cases, translation initiation could occur at an alternative start codon located in-frame but farther downstream, which would place these TSS within the upstream intergenic region (Additional file 4: Table S4). As an example, TK1361 is annotated as encoding a mini-chromosome maintenance protein (MCM2) with an atypically long N-terminal extension [57, 58]. In the dRNA-seq data, an iTSS is present within TK1361 and transcripts initiated at this site would have a RBS and encode a standard MCM with no extension (Figure 5A). Interestingly, TK1620 that also encodes a MCM homologue (MCM3), also contains an iTSS positioned appropriately downstream of a BRE-TATA-box sequence and upstream of a potential RBS and an in-frame GTG start codon, although translation initiated at this GTG would result in a much truncated MCM3. TK1620 is also apparently the promoter distal gene in a TK1619-TK1620 operon for which a well-defined pTSS was identified (Figure 5B). The MCM3 protein is synthesized initially as a precursor, containing a homing-endonuclease intein and as the iTSS, its putative promoter and the downstream GTG codon are all within the intein-encoding region, these regulatory elements and/or the truncated MCM3 may participate in MCM3 maturation (Figure 5B).

For all but three of the remaining iTSS, an appropriately positioned BRE-TATA box sequence is readily apparent consistent with the downstream region of the ORF being transcribed from two structurally separate promoters. In some, but not all cases, there are also sequences that could function as a RBS and translation initiating codon, within the ORF, downstream of the iTSS. In the absence of such translation initiating elements, transcription from the iTSS presumably results in a non-coding RNA as exemplified by the HgcC transcripts. HgcC RNAs were originally identified by bioinformatically and their expression then verified by northern blot analysis [56]. As documented in the RFAM database, homologous sequences are found in 43 *Archaea*, most are hyperthermophiles, but some are halophiles [38]. The *T. kodakarensis* genome has sequences encoding seven HgcC transcripts [36] and the dRNA-seq data confirm that five of these, designated TK HgcC_1_ to HgcC_5_, are present in *T. kodakarensis* cells (Additional file 6: Figure S2). As reported for an HgcC in *P. furiosus*[56], TK HgcC_1_, TK HgcC_2_ and TK HgcC_3_ are transcribed from transposase encoding sequences. The *P. furiosus* HgcC transcript is, however, an antisense transcript relative to the transposase gene whereas TK HgcC_1_, HgcC_2_ and HgcC_3_ are transcribed in the same direction from iTSSs within the transposase genes, TK0298, TK0495 and TK0850, respectively (Additional file 7: Figure S3A-C). TK HgcC_4_ and TK HgcC_5_ are not associated with transposase genes. HgcC_5_ has an iTSS within TK1820 (membrane-associated metalloprotease), and the TSS of HgcC_4_ is also the pTSS of TK1679 as HgcC_4_ is encoded within the 5′-region of TK1679 (hypothetical protein) (Additional file 7: Figure S3D-E). An antisense RNA, complementary to part of HgcC_4_, is also transcribed from the TK1820 region that could function in *trans* as a regulator of HgcC synthesis and/or function (Additional file 7: Figure S3E).

### Antisense transcripts in *T. kodakarensis*

Before the advent of deep sequencing, most non-coding RNAs were identified through sequence conservation and predictions for conserved RNA folding, with screening for non-coding RNAs generally avoiding ORFs and their complementary antisense regions. However, as transcriptome data have accumulated, it has become increasingly clear that antisense transcription is a major feature of prokaryotic and eukaryotic genome expression [59]. The dRNA-seq data identified 1,018 aTSS, sites at which antisense transcription is initiated on the *T. kodakarensis* genome. The aTSS are not evenly distributed. Inspection of 150 bp windows around translation start and stop codons revealed that 58% of the aTSS are located near gene termini with 260 and 329 antisense transcripts overlapping the 5′- and 3′-terminus, respectively, of an ORF. A similar enrichment of antisense transcription across gene termini has also been observed in other *Archaea*, with antisense transcripts often associated with transposase-encoding genes [21, 22, 25, 27, 30, 31, 60–63]. An aTSS is associated with all seven genes (TK0298, TK0495, TK0654, TK0850, TK0931, TK0932, TK1842) annotated as encoding transposases in the *T. kodakarensis* genome [37]. To identify any additional preferential associations of aTSS with specific functions, the aTSS locations were evaluated relative to the pathways and functions defined in the KEGG database [64, 65]. Antisense transcripts are associated with 19, 14, 13 and 12 ORFs that encode proteins involved in amino acid, purine, pyrimidine and central carbon metabolism, respectively, 12 that participate in ribosome biogenesis and 7 that encode ABC transporters. At least some of these antisense RNAs most likely interact with the complementary sense mRNA but, as yet, there is no direct experimental evidence for such an interaction *in vivo*. This has been documented experimentally *in vitro* for an antisense RNA from *M. mazei* that sequestered the RBS of the target mRNA [66].

### Known or predicted small non-coding RNAs

The RFAM and UCSC databases predict that ~500 small non-coding RNAs are encoded in the *T. kodakarensis* genome [38, 39]. Most are classified as snoRNAs, archaeal counterparts of eukaryotic snoRNAs (small nucleolar RNAs) that direct 2′-O-methylation (C/D box) and pseudouridylation (H/ACA box) of transfer (tRNA) and ribosomal RNAs (rRNA). We verified the presence of 69 of these small non-coding RNAs (Additional file 8: Table S5); 54 designated as C/D box and 7 as H/ACA box snoRNAs. Of these, 17 are not recognizably linked to annotated genes and so are classified as orphan snoRNAs. There are 11 potential snoRNAs encoded within 5′-UTRs and 12 within the 5′-coding region of ORFs that, presumably, must be released by transcript processing. Tko-sR44, for example, appears to be co-transcribed with a tRNA^Arg^ and is then likely released from the co-transcript by RNase Z cleavage, as observed in *Nanoarchaeum equitans* and in some plants [67, 68]. In some cases, the presence of a K-turn motif near the 5′-terminus, the region predicted to be a snoRNA may, in fact, be a *cis*-regulatory element rather than a snoRNA. K-turn motifs are important structural elements in riboswitches [69, 70]. Alternatively, as sequences that conform to ribosomal protein L7Ae (TK1311) binding sites are present in several of these transcripts, including those encoding the aNOP56 (TK0184) and Cbf5p (TK1509) components of the snoRNA guide complexes, these transcripts might be processed by complexes containing L7Ae and possibly RNase P [71]. Seventeen of the potential snoRNAs are antisense transcripts, 16 of which are transcribed from DNA that includes the 3′-terminus of the complementary sense gene. In 7 cases, a snoRNA is transcribed convergent to an antisense RNA, a gene organization also documented in *S. solfataricus, N. equitans* and *Pyrobaculum* species [22, 25, 28, 62].

Deep sequencing identified 107 sense and 215 antisense non-coding RNAs in *P. abyssi* GE5 [31]. Based on a *BLAST* search, 68 of these are also encoded in the *T. kodakarensis* genome, of which 33 are clearly represented in the dRNA-seq libraries. A further 8 orphan small non-coding RNAs predicted and/or documented to be present in *Pyrococcus* species [23, 31, 56] (Additional file 9: Table S6) and the small non-coding RNAs, designated CRISPR RNAs (crRNA), predicted to be transcribed from the three CRISPR loci in the *T. kodakarensis* genome are also present in the *T. kodakarensis* dRNA-seq libraries [37, 56]. Transcription of a CRISPR locus generates a long transcript that is cleaved, first releasing immature crRNAs with 8 nt 5′-extensions and variable 3′-termini. These are then trimmed to produce the mature crRNAs. The TSS identified are fully consistent with the locations predicted for promoters within the CRISPR loci and with this transcript processing [72].

### Orphan small non-coding RNAs

In addition to the 17 orphan snoRNAs detected and the non-coding small RNAs also identified in *Pyrococcus* species (see above), the dRNA-seq libraries document the presence of 55 previously unrecognized small transcripts, including an unrecognized snoRNA (Additional file 8: Table S5). ORFs >10 codons are not present in any of these transcripts and only 14 (26%) are conserved in other *Archaea* (Additional file 9; [21, 22, 27]) arguing for *T. kodakarensis* specific functions. Historically, computer screening for non-coding RNAs in hyperthermophiles has employed a high GC content (>50% GC) as a filter [56, 73] but only 30% of the non-coding RNAs now identified in *T. kodakarensis* have GC contents >50% and only 9 of those first reported here meet this criterion. Given that the sequences of these transcripts are, on average ~43% GC, they seem unlikely to have stable base-paired secondary structures *in vitro* at the 85°C optimum growth temperature of *T. kodakarensis* but may have secondary structures stabilized *in vivo* by protein interactions. This is predicted for Lsm binding to several sRNAs in *H. volcanii*[74] and the *T. kodakarensis* genome encodes several RNA-binding proteins [37], in addition to Lsm, with Alba (TK0570) and a putative RNA-binding protein encoded by TK2065 likely present in very high abundance (Table 1 and Additional file 10: Table S7).

**Table 1:** **Protein-encoding genes with the highest numbers of reads per ORF in the pyruvate (P**_**exp**_ **) and sulfur (S**_**exp**_ **) cDNA libraries**

	^1^ TK#	P _exp_ library	TK#	S _exp_ library
1	108^2^	Tko-sR04 + hypothetical protein	1311	50S ribosomal protein L7 Ae
2	1311	50S ribosomal protein L7 Ae	560	DNA/RNA-binding protein A1bA
3	560	DNA/RNA-binding protein A1bA	1694	ferrodoxin 1
4	1694	ferredoxin 1	108^2^	Tko-sR04 + hypothetical protein
5	1067	hypothetical protein	2289	histone B
6	2289	histone B	1417	50S ribosomal protein L1P
7	1431	glutamate dehydrogenase	1411	hypothetical protein
8	1331	Lrp/AsnC family transcriptional regulator	1431	glutamate dehydrogenase
9	1484	hypothetical protein	895	S-layer protein
10	895	S-layer protein	1331	Lrp-AsnC family transcriptional regulator
11	1411	hypothetical protein	1416	Acidic ribosomal protein P0
12	2284^2^	Tko-sR67 + 7, 8-dihydro-8-oxoguanine-triphosphatase	2286	H/ACA RNA protein complex Garl
13	537	peroxiredoxin	1067	hypothetical protein
14	1417	50S ribosomal protein L1P	1484	hypothetical protein
15	2065	RNA-binding protein	594	hypothetical protein
16	1245	hypothetical protein	1251	30S ribosomal protein S15
17	1416	acidic ribosomal protein P0	2065	RNA-binding protein
18	1565^2^	Tko-sR50 + hypothetical protein	1245	hypothetical protein
19	36	hypothetical protein	2284^2^	Tko-sR67 + 7, 8-dihydro-8-oxoguanine-triphosphatase
20	38	flagellin	615	50S ribosomal protein L37 Ae
21	2006	hypothetical protein	904	hypothetical protein
22	594	hypothetical protein	738	hypothetical protein
23	1651	hypothetical protein	1016	hypothetical protein
24	1842	Transposase	537	peroxiredoxin
25	1004	UDP-glucose 4-epimerase	1543	hypothetical protein

^1^The TKxxxx numerical gene designation for the promoter proximal gene in the transcription units that had ≥4,000 reads (Additional file 10: Table S7). The genes are listed in decreasing order of cDNA reads, with shading identifying genes present in both lists.

^2^The automated analysis assigned the cDNA reads to genes TK0108, TK2284 and TK1565. Manual inspection revealed the presence of Tko-sR04, Tko-sR67 and Tko-sR50, respectively, within the 5′-terminal region of each transcript.

### tRNAs, rRNAs, RNase P and 7S RNAs

All of the tRNAs and rRNAs annotated in the *T. kodakarensis* genome are present and fully covered in the dRNA-seq libraries (Additional file 11: Table S8). There are also antisense transcripts present complementary to six tRNAs and one tRNA^Thr^ is, itself, an antisense transcript of TK1287 (encodes uracil phosphoribosyltransferase). As described above, one tRNA^Arg^ appears to be cotranscribed with a snoRNA (Tko-sR44) and a snoRNA (designated Tko-19) encoding sequence is located immediately upstream, and is likely within the 5′-leader region and so cotranscribed with the 16S rRNA-tRNA^Ala^-23S rRNA operon (Additional file 12: Figure S4A)

The dRNA-seq libraries also confirm the presence of the RNase P RNA and 7S (SRP) RNA in *T. kodakarensis* cells although the 7S RNA is transcribed from the DNA strand opposite to that stated in the genome annotation [37]. The dRNA-seq data are convincing (Additional file 12: Figure S4B) and in agreement with the RFAM database [38].

### Growth and media-dependent transcription

Based on previous studies of *P. furiosus*[32, 33] and *T. kodakarensis*[34, 35], we generated cDNA libraries from cells growing in media with sulfur (S_exp_) or pyruvate (P_exp_) to increase the number of TSS likely identified. We also generated cDNA libraries from cells grown with sulfur to stationary phase (S_stat_) and from cells growing with pyruvate but with sulfur added 20 min before RNA isolation (PS). The RNA preparations were subjected to TEX digestion before cDNA synthesis and, given *in vivo* transcript processing and *in vitro* fragmentation during purification, the resulting cDNA libraries were, as expected, enriched for 5′-terminal sequences. This facilitates the identification of TSS but, assuming that the number of cDNA reads correlates with transcript abundance, the dRNA-seq data also provide a semi-quantitative overview of global genome expression and are consistent with previous observations of substrate-dependent specific gene expression. Based on the number of cDNA reads, there is little or no transcription from ~35% of transcriptional units (TUs) in cells growing in pyruvate medium, of ~28% of the TUs in cells growing in sulfur medium, and of ~87% of TUs in stationary phase cells. Transcript abundances vary substantially, but <2.5% are present >1000-fold above the minimal detectable level (Additional file 13: Table S9). Consistent with constitutively high expression and/or transcript stability, 18 of the 25 most abundant transcripts were the same in RNA preparations from cells growing in sulfur or pyruvate medium (Table 1)

**Figure 6:**
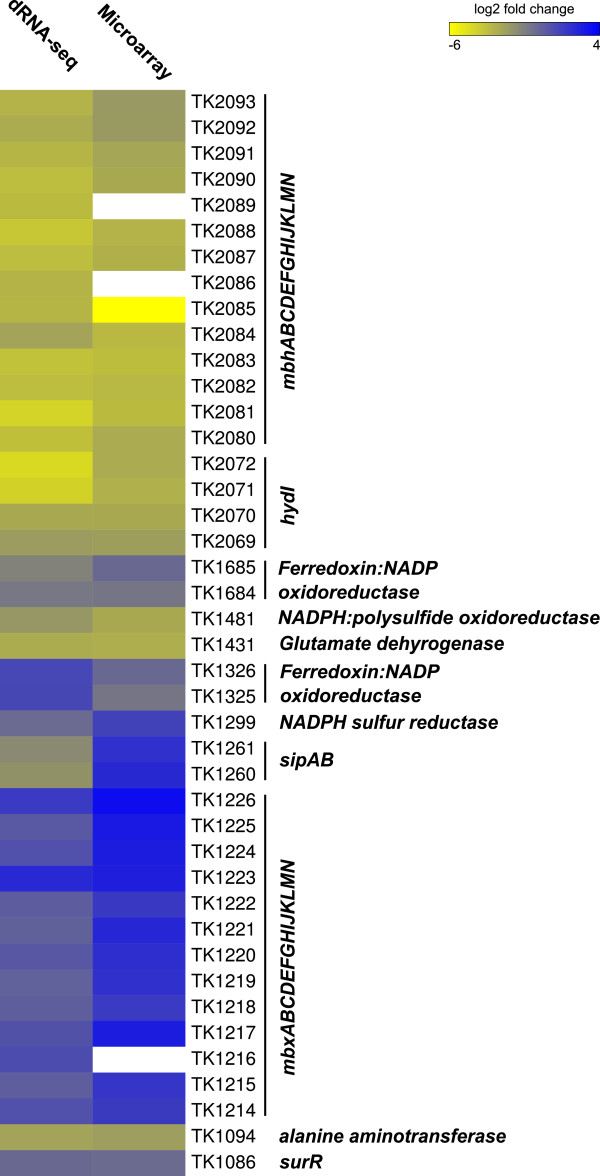
**Heatmap comparison of changes in gene expression after sulfur addition.** Changes in transcript abundance are shown, on a log2-fold scale, for the TK genes listed. Comparisons were made of the cDNA libraries generated from RNA isolated before and 20 min after addition of sulfur to *T. kodakarensis* cells growing exponentially in pyruvate. Values were calculated based on changes in the abundance of cDNA reads, as described in Materials and Methods. The *T. kodakarensis* data are aligned and compared with the microarray hybridization results reported for sulfur-induced changes in transcription of the homologous *mbx* and *mbh* operons, and related homologous genes, in *P. furiosus*[33].

Schut et al [33] documented that when sulfur was added to *P. furiosus* cultures growing in pyruvate medium, H_2_S replaced H_2_ as an end-product of metabolism and transcription of the *mbh* operon was replaced by transcription of the *mbx* operon. This metabolic shift also occurs in *T. kodakarensis*[34, 35] and, based on the numbers of cDNA reads obtained from RNA preparations isolated from *T. kodakarensis* cells growing in pyruvate medium before and after sulfur addition, the transcription regulation is also conserved in *T. kodakarensis*. As in *P. furiosus*[33], transcription of the *mbh* operon (TK2080 -TK2093) that encodes the H_2_-generating hydrogenase is rapidly and almost fully terminated following sulfur addition and replaced by transcription of the *mbx* operon (TK1226 - TK1214) that encodes the H_2_S-generating enzyme. (Figure 6).

The reductant needed to generate H_2_ and/or H_2_S is most likely supplied by a reduced ferredoxin [34, 35] but there are three candidate ferredoxins encoded in the *T. kodakarensis* genome [38]. Based on cDNA reads, transcripts of TK1694 (encodes ferredoxin-1) are very abundant under all of the growth conditions investigated (Table 1 and Additional file 10: Table S7), indicative of ferredoxin-1 participating in many metabolic pathways, although there is a ~2-fold decrease after sulfur addition to pyruvate growing cells. Ferredoxin-2 is encoded by TK1087, the middle gene in a three gene operon (TK1086-TK1088) that also encodes SurR (TK1086), a redox-responsive transcription regulator of many genes involved in sulfur metabolism [75]. The extent of ferredoxin-2 reduction could provide redox-state information to SurR, and so modulate SurR activity, but sulfur addition had little effect on the abundance of TK1086-TK1088 transcripts (Additional file 10: Table S7). In contrast, there was a ~6-fold decrease in TK2012 transcripts (encodes ferredoxin-3) following sulfur addition to pyruvate growing cells, arguing that ferredoxin-3 is likely the predominant electron donor for H_2_ production by the *mbh* encoded hydrogenase (Additional file 10: Table S7). Intriguingly, an antisense transcript is also generated from the TK2012 region that has increased expression in the presence of sulfur (Additional file 14: Figure S5), and a SurR binding site overlaps the TATA box of the promoter for this antisense transcript. The antisense RNA is therefore likely part of the SurR regulon, and TK2012 expression and so ferredoxin-3 synthesis may be indirectly subject to SurR regulation through post-transcription regulation by the antisense RNA.

## Discussion and conclusions

The results obtained establish that transcription initiation occurs at >2,700 sites around the *T. kodakarensis* genome (Additional file 2) and recognizable BRE-TATA-box promoter elements are appropriately located upstream of ~78% of all the *T. kodakarensis* transcription units identified. As reported for other *Archaea*[22, 25, 51, 56, 76], many promoters and TSS are embedded within ORFs, and antisense transcription occurs extensively throughout the *T. kodakarensis* genome adding significantly to the genome complexity, and predicting a major involvement of antisense transcripts in regulating gene expression. *T. kodakarensis* cells contain many small non-coding RNAs, some previously identified or predicted including two candidate riboswitches [23, 31, 51, 56] but also many previously unanticipated RNAs (Additional file 8: Table S5, Additional file 9: Table S6 and Additional file 10: Table S7). As in *P. abyssi*[31], some have relatively AU-rich sequences, in contrast to the high GC content of *T. kodakarensis* tRNAs and rRNAs, and contrary to the expectation [56, 73] that small non-coding RNAs in hyperthermophiles would be GC-rich to stabilize secondary structures. The sequences of most of these small AU-rich RNAs do not readily fold into canonical base-paired secondary structures, and may function as unstructured molecules [31], but could have secondary and tertiary structures stabilized in vivo by nucleic acid and protein interactions [74]. Most 5′-UTRs in *T. kodakarensis* are short and some are leaderless (Figure 1). But, as in other *Archaea*[21, 22, 32], there are also mRNAs with long 5′-UTRs that are consistent and predictive of *cis*-regulatory elements, although there is no direct experimental support to date for regulation *in vivo* by attenuators or riboswitches in *Archaea*.

The most exciting and experimentally-challenging conclusion from this, and from all other archaeal transcriptome studies to date, is that archaeal cells contain many different, often abundant and apparently non-coding small RNAs. *T. kodakarensis* appears typical; it has a very small genome (~2.1 Mbp) tightly packed with ORFs but also with genes that encode non-translated RNAs and so likely has widespread RNA-based regulation. Historically, RNA secondary structure was sought and equated with non-coding RNA function but it is now clear that such structure is not mandatory. For example eukaryotic siRNAs and lncRNAs apparently bind their RNA and protein targets, and carry out their regulatory functions, without extensive structure [77, 78]. Given that gene expression in *Archaea,* and particularly transcription-related features are simpler but have many features in common with their eukaryotic counterparts, it seems likely that investigating RNA-based regulation in *Archaea,* with *T. kodakarensis* providing a model system [3], will generate results that are valuable and legitimately extrapolated into eukaryotic gene expression.

## Methods

### *T. kodakarensis*growth

*T. kodakarensis* cultures were grown anaerobically at 85°C in nutrient-rich artificial sea water medium that contained 5 g/l yeast extract, 5 g/l tryptone (ASW-YT), the required trace minerals and vitamins [34, 79], and either 2 g S°/l (Sulfur medium) or 5 g sodium pyruvate/l (Pyr medium). The growth of cultures was followed by optical density measurements at 600 nm (OD_600_) and, in most experiments, aliquots (50-500 ml) were removed for RNA isolation when the OD_600_ reached 0.2. In experiments where sulfur was added (final concentrations of 2 g/l) to cultures growing in Pyr medium, the addition occurred when the culture reached an OD_600_ of 0.2, and a 500 ml aliquot was removed for RNA isolation after a further 20 min incubation at 85°C.

### RNA extraction

Cells were removed from suspension by centrifugation (4000 g; 30 min) at 4°C, the resulting cell pellet immediately resuspended in TRIzol (Invitrogen), instantly frozen in liquid nitrogen and stored at -70°C. After thawing, total RNA was extracted using the TRIzol manufacturer’s protocol, then incubated at 37°C for 1 h with DNAse I (Thermo Fisher Scientific Inc) and an aliquot subjected to agarose gel electrophoresis and visualized by staining to determine the size-profile of the RNA molecules present. The concentration of the RNA solution was determined using a NanoDrop 1000 spectrophotometer (Thermo Fischer Scientific Inc).

### Construction of cDNA libraries and Illumina sequencing

The cDNA libraries were constructed as previously described [7, 15]. For Illumina sequencing (HiSeq) of cDNA molecules, the libraries were constructed by *vertis* Biotechnology AG, Germany, as described previously for eukaryotic microRNA libraries [80] but without a RNA size-fractionation step before the cDNA synthesis. The cDNA libraries were sequenced using a HiSeq 2000 machine (Illumina) in single-read mode and 100 cycles. The raw, de-multiplexed reads and coverage files have been deposited in the National Center for Biotechnology Information Gene Expression Omnibus (NCBI GEO; [81]) with accession code GSE56262. Detailed descriptions of procedures used for read mapping, expression graph construction and normalization of expression graphs have been published [15]. For graph visualization the Integrative Genomics Viewer (IGV version 2.3.27) was used [82].

### Transcriptional start site (TSS) annotation and expression analysis

The pooled sequence reads were de-multiplexed and the adapter sequences were removed. The reads in Fastq format were then quality trimmed using *fastq_quality_trimmer* (FastX suite version 0.0.13 [83]) with a cut-off Phred score of 20 and converted to Fasta format using *fastq_to_fasta* (FastX suite). The read processing [including poly(A) removal, size filtering (min 12 nt length), statistics generation, coverage calculation and normalization] was performed using the RNA-analysis pipeline *READemption* version 0.1.6 [84] with default parameters which used *segemehl* version 0.1.4 [35]. An automated pipeline (TSSpredator) was used to identify the TSS [15]. The software was provided with the *T. kodakarensis* genome annotation [37] extended by entries of known and predicted RNAs taken from the RFAM database [38] and the UCSC archaeal genome browser [39]. TSS were first identified in the cDNA libraries (S_exp_ and C), generated with and without TEX digestion, and the remaining libraries were then manually checked to confirm these TSS and for additional TSS. As illustrated (Figure 1A), the TSS were defined and grouped as primary (pTSS), secondary (sTSS), internal (iTSS) and/or antisense (aTSS) transcription start sites, depending on their location relative to an annotated gene. Based on the location of a translation start codon, the distribution of the lengths predicted for 5′-UTRs was visualized using *RStudio* (RStudio, Inc.) and the *ggplot2* package [85]. The bioconductor package *DEseq*[86] was used to measure expression with the results listed in Additional file 10: Table S7. A heatmap comparison (Figure 6) of dRNA-seq data from *T. kodakarensis* and microarray expression data for *P. furiosus*[32] taken from the NCBI GEO database (GPL4688) was generated using *heatmap.plus*. The organization of genes into transcription units (operons) in the *T. kodakarensis* genome was taken from the DOOR^2^ database [41], and to calculate transcript abundances, all ORFs in an operon were grouped and the normalized average reads per gene (Additional file 10: Table S7) were summed (Additional file 13: Table S9).

### Promoter and RBS motifs detection and data visualization

To identify promoter motifs, the sequences from 50 bp upstream of each TSS to the TSS were scanned by *MEME* version 4.8.1 [46] using standard parameters, but searching only the sense strand. Ribosome binding sites (RBS) were located in mRNAs by *MEME* and potential RBS in previously unrecognized transcripts were sought by *FIMO* using standard settings [46] and the *MEME* generated position weight matrix (PWM) as input (Additional file 15). When a TSS indicated a leaderless mRNAs, a 100 bp window around the TSS was scanned with *FIMO* and all alternative translation initiation sites so detected were manually inspected. When deemed likely, a start codon was reassigned (Additional file 4: Table S4) and the TSS then, as necessary, re-categorized.

### Conservation of small non-coding RNAs

The RFAM 11.0 database [38] was screened using c*msearch* of the *INFERNAL* package version 1.1 with standard settings [87] to detect all known sRNAs. *snoScan*[88] and *snoReport*[89] were applied to identify additional potential snoRNAs within the previously unrecognized sRNAs. To identify sRNA homologues, the NCBI nucleotide database restricted to the domain *Archaea* was searched using *blastn* (part of *BLAST+*, version 2.2.28 [90]). The word-size parameter was set to 10 nt, an empirical filter used to identify *blastn* alignments with an expected value (e-value) < 0.06, and all potential homologues were then manually inspected. The number of identical nucleotides in sRNA alignments was divided by the total number of nucleotides in the query sRNA, and multiplied by 100 to obtain a percentage conservation value. Only conservation values ≥40% were retained for further analysis (Additional file 9: Table S6). The extent of conservation, determined by a BLAST analysis, is given as the closest common taxonomic level. RNA secondary structure predictions were performed using *RNAfold* (*ViennaRNA packag*e version 2.1.g [91]). Orphan transcripts were screened for ORFs and all putative polypeptides containing at least 20 amino acid residues were used as query proteins in *blastp* analyses (part of *BLAST+*, version 2.2.28 [90]) with default parameters. Only homology pairs with an e-value < 10^-3^ were further considered.

## Electronic supplementary material

Additional file 1: Table S1: Mapping statistics of *T. kodakarensis* dRNA-seq libraries. The Table lists the total number of sequenced cDNA reads considered in the analysis, the number of reads that were removed due to insufficient length (<12 nt) after poly(A)-tail trimming (before read mapping), the number of reads that were successfully mapped to the reference genome, the number of mappings, and the number of uniquely mapped reads. Percentage values (relative to the total number of reads) are also provide for the number of mapped reads and number of uniquely mapped reads. (XLSX 369 KB)

Additional file 2: Table S2: list of all identified TSS. The Table provides the positions and assigned classes of all identified TSS. When a TSS is assigned to more than one category, there is one row for each assignment with the associated gene. (XLSX 147 KB)

Additional file 3: Table S3: Previously experimentally determined TSS. The Table lists all previously known or inferred TSS in *T. kodakarensis* and relates them to the dRNA-seq identified TSS. (XLSX 7 KB)

Additional file 4: Table S4: List of likely mis-annotated genes. The Table lists positions of TSS and the corrected translational start sites of genes that were considered mis-annotated. (XLSX 10 KB)

Additional file 5: Figure S1: Sequences upstream of sRK28 and TK1195. The sequences upstream of sRK28 and TK1195 and sRk28 are shown with the promoter elements (BRE; TATA-box), and the TSS (+1) documented by dRNA-seq, identified in bold text. The region encoding sRK28 is highlighted in red. The sequence encoding the 5′-UTR (grey) and the GTG translation initiating codon of TK1195 are identified. (PDF 23 KB)

Additional file 6: Figure S2: Secondary structure predictions of TEX resistant regions of transcripts from near the 3′-termini of genes. The sequences shown, transcribed from regions near the 3′-termini of the indicated genes, survived TEX digestion and so were prevalent in the dRNA-seq libraries. The secondary structures shown, and predicted stabilities, were generated by *RNAfold* 91. (PDF 32 KB)

Additional file 7: Figure S3: HgcC transcripts encoded in the *T. kodakarensis* genome. A-E. The genomic locations of five TK HgcC transcripts (black boxes). The abundances of transcripts synthesized from these regions in *T. kodakarensis* cells growing exponentially (S_exp_; blue) and in stationary phase (S_stat_; red) in sulfur medium, growing exponentially in pyruvate medium before (P_exp_; green) and 20 min after sulfur addition (PS; orange) are given by the peak heights. Data from the control library (C) not digested with TEX are shown in grey. As illustrated, an antisense RNA is also transcribed from the region encoding HgcC_5_. The relative abundance scales on the right of each panel allow direct comparisons of all data in that panel. The black scale bar in the top right corner of each panel is corresponds to 100 nt. (PDF 1 MB)

Additional file 8: Table S5: snoRNAs in the *T. kodakarensis* genome. The Table lists all candidate snoRNAs identified in the *T. kodakarensis* genome. (XLSX 9 KB)

Additional file 9: Table S6: Small non-coding RNAs in the *T. kodakarensis* genome. The Table lists all candidate sRNA identified in the *T. kodakarensis* genome. (XLSX 10 KB)

Additional file 10: Table S7: Expression data for detected genes in all dRNA-seq libraries. The Table lists the average normalized read countings obtained by dRNA-seq and the *DEseq* derived expression data calculated as described in Methods. (XLSX 397 KB)

Additional file 11: Table S8: tRNA, rRNA and other well documented non-coding RNAs in *T. kodakarensis.* The Table lists all tRNAs, rRNAs and other well documented stable RNAs encoded in the *T. kodakarensis* genome and identifies their TSS. (XLSX 10 KB)

Additional file 12: Figure S4: Transcription of the rRNA operon and SRP RNA. (A) As illustrated, the 16S-tRNA^Ala^-23S rRNA operon is cotranscribed with a snoRNA, designated Tko19. (B) The SRP RNA is transcribed from the stand opposite that designated in the genome annotation [38]. The abundances of transcripts present in cells growing exponentially (S_exp_; blue) and in stationary phase (S_stat_; red) in sulfur medium, growing exponentially in pyruvate medium before (P_exp_; green) and 20 min after sulfur addition (PS; orange) are given by the peak heights. Data from the control library (C) not digested with TEX are shown in grey. The relative abundance scales on the right of each panel allow direct comparisons of all data in that panel. The black scale bar in the top right corner of each panel is corresponds to 100 nt. (PDF 832 KB)

Additional file 13: Table S9: Read count percentage of transcriptional units. Genes were grouped into transcription units according to the DOOR^2^ database [40 41] and the average normalized read countings (Additional file 10) for each transcription unit were summed. The percentage of the 1,290 transcription units [40, 41] with numbers of reads in the indicated range are given for the sulfur (S_exp_ and S_stat_) and pyruvate (P_exp_) libraries. (XLSX 8 KB)

Additional file 14: Figure S5: Genome organization around TK2012 (encodes ferredoxin-3). As illustrated, a putative SurR binding site (red font) overlaps the BRE-TATA-box region of a promoter that directs transcription of an antisense RNA (black box) from the TK2012 region. The abundances of transcripts present in cells growing exponentially (S_exp_; blue) and in stationary phase (S_stat_; red) in sulfur medium, growing exponentially in pyruvate medium before (P_exp_; green) and 20 min after sulfur addition (PS; orange) are given by the peak heights. Data from the control library (C) not digested with TEX are shown in grey. The relative abundance scales on the right of each panel allow direct comparisons of all data in that panel. The black scale bar in the top right corner of each panel is corresponds to 100 nt. (PDF 309 KB)

Additional file 15: **Positions weight matrix (PWM) used for the identification of RBS in the dRNA-seq data.** Ribosome binding sites (RBS) were located in all annotated mRNAs by *MEME*[45]*. A* 10 nt window upstream of every start codon was extracted and scanned. The generated PWM was used as input for *FIMO*[46]. (ZIP 506 bytes)

## References

[1] Cavicchioli R (2011). Archaea–timeline of the third domain. Nat Rev Microbiol.

[2] Sato T, Fukui T, Atomi H, Imanaka T (2003). Targeted gene disruption by homologous recombination in the hyperthermophilic archaeon thermococcus kodakarensis KOD1. J Bacteriol.

[3] Farkas JA, Picking JW, Santangelo TJ (2013). Genetic techniques for the archaea. Annu Rev Genet.

[4] Wang Z, Gerstein M, Snyder M (2009). RNA-Seq: a revolutionary tool for transcriptomics. Nat Rev Genet.

[5] Croucher NJ, Thomson NR (2010). Studying bacterial transcriptomes using RNA-seq. Curr Opin Microbiol.

[6] Berghoff BA, Glaeser J, Sharma CM, Vogel J, Klug G (2009). Photooxidative stress-induced and abundant small RNAs in rhodobacter sphaeroides. Mol Microbiol.

[7] Sharma CM, Hoffmann S, Darfeuille F, Reignier J, Findeiss S, Sittka A, Chabas S, Reiche K, Hackermüller J, Reinhardt R, Stadler PF, Vogel J, Hackermuller J (2010). The primary transcriptome of the major human pathogen helicobacter pylori. Nature.

[8] Irnov I, Sharma CM, Vogel J, Winkler WC (2010). Identification of regulatory RNAs in bacillus subtilis. Nucl Acids Res.

[9] Albrecht M, Sharma CM, Reinhardt R, Vogel J, Rudel T (2010). Deep sequencing-based discovery of the chlamydia trachomatis transcriptome. Nucl Acids Res.

[10] Vockenhuber M-P, Sharma CM, Statt MG, Schmidt D, Xu Z, Dietrich S, Liesegang H, Mathews DH, Suess B (2011). Deep sequencing-based identification of small non-coding RNAs in streptomyces coelicolor. RNA Biol.

[11] Mitschke J, Georg J, Scholz I, Sharma CM, Dienst D, Bantscheff J, Voss B, Steglich C, Wilde A, Vogel J, Hess WR (2011). An experimentally anchored map of transcriptional start sites in the model cyanobacterium synechocystis sp. PCC6803. Proc Natl Acad Sci USA.

[12] Mraheil M a, Billion A, Mohamed W, Mukherjee K, Kuenne C, Pischimarov J, Krawitz C, Retey J, Hartsch T, Chakraborty T, Hain T (2011). The intracellular sRNA transcriptome of Listeria monocytogenes during growth in macrophages. Nucl Acids Res.

[13] Arnvig KB, Comas I, Thomson NR, Houghton J, Boshoff HI, Croucher NJ, Rose G, Perkins TT, Parkhill J, Dougan G, Young DB (2011). Sequence-based analysis uncovers an abundance of non-coding RNA in the total transcriptome of Mycobacterium tuberculosis. PLoS Pathog.

[14] Schmidtke C, Findeiss S, Sharma CM, Kuhfuss J, Hoffmann S, Vogel J, Stadler PF, Bonas U (2012). Genome-wide transcriptome analysis of the plant pathogen Xanthomonas identifies sRNAs with putative virulence functions. Nucl Acids Res.

[15] Dugar G, Herbig A, Förstner KU, Heidrich N, Reinhardt R, Nieselt K, Sharma CM (2013). High-resolution transcriptome maps reveal strain-specific regulatory features of multiple campylobacter jejuni isolates. PLoS Genet.

[16] Toffano-Nioche C, Nguyen AN, Kuchly C, Ott A, Gautheret D, Bouloc P, Jacq A (2012). Transcriptomic profiling of the oyster pathogen Vibrio splendidus opens a window on the evolutionary dynamics of the small RNA repertoire in the Vibrio genus. RNA.

[17] Soutourina O a, Monot M, Boudry P, Saujet L, Pichon C, Sismeiro O, Semenova E, Severinov K, Le Bouguenec C, Coppée J-Y, Dupuy B, Martin-Verstraete I (2013). Genome-wide identification of regulatory RNAs in the human pathogen Clostridium difficile. PLoS Genet.

[18] Mentz A, Neshat A, Pfeifer-Sancar K, Pühler A, Rückert C, Kalinowski J (2013). Comprehensive discovery and characterization of small RNAs in Corynebacterium glutamicum ATCC 13032. BMC Genomics.

[19] Madhugiri R, Pessi G, Voss B, Hahn J, Sharma CM, Reinhardt R, Vogel J, Hess WR, Fischer H-M, Evguenieva-Hackenberg E (2012). Small RNAs of the Bradyrhizobium/Rhodopseudomonas lineage and their analysis. RNA Biol.

[20] Phillips P, Progulske-Fox A, Grieshaber S, Grieshaber N (2014). Expression of Porphyromonas gingivalis small RNA in response to hemin availability identified using microarray and RNA-seq analysis. FEMS Microbiol Lett.

[21] Jäger D, Sharma CM, Thomsen J, Ehlers C, Vogel J, Schmitz RA (2009). Deep sequencing analysis of the Methanosarcina mazei Go1 transcriptome in response to nitrogen availability. Proc Natl Acad Sci USA.

[22] Wurtzel O, Sapra R, Chen F, Zhu Y, Simmons BA, Sorek R (2009). A single-base resolution map of an archaeal transcriptome. Genome Res.

[23] Phok K, Moisan A, Rinaldi D, Brucato N, Carpousis AJ, Gaspin C, Clouet-d’Orval B (2011). Identification of CRISPR and riboswitch related RNAs among novel noncoding RNAs of the euryarchaeon Pyrococcus abyssi. BMC Genomics.

[24] Bernick DL, Dennis PP, Höchsmann M, Lowe TM (2012). Discovery of Pyrobaculum small RNA families with atypical pseudouridine guide RNA features. RNA.

[25] Bernick DL, Dennis PP, Lui LM, Lowe TM (2012). Diversity of antisense and other non-coding RNAs in Archaea revealed by comparative small RNA sequencing in four Pyrobaculum species. Front Microbiol.

[26] Danan M, Schwartz S, Edelheit S, Sorek R (2012). Transcriptome-wide discovery of circular RNAs in archaea. Nucl Acids Res.

[27] Heyer R, Dörr M, Jellen-Ritter A, Späth B, Babski J, Jaschinski K, Soppa J, Marchfelder A (2012). High throughput sequencing reveals a plethora of small RNAs including tRNA derived fragments in Haloferax volcanii. RNA Biol.

[28] Randau L (2012). RNA processing in the minimal organism Nanoarchaeum equitans. Genome Biol.

[29] Xu N, Li Y, Zhao Y-T, Guo L, Fang Y-Y, Zhao J-H, Wang X-J, Huang L, Guo H-S (2012). Identification and characterization of small RNAs in the hyperthermophilic archaeon Sulfolobus solfataricus. PLoS One.

[30] Su AH, Tripp V, Randau L (2013). RNA-Seq analyses reveal the order of tRNA processing events and the maturation of C/D box and CRISPR RNAs in the hyperthermophile Methanopyrus kandleri. Nucl Acids Res.

[31] Toffano-Nioche C, Ott A, Crozat E, Nguyen AN, Zytnicki M, Leclerc F, Forterre P, Bouloc P, Gautheret D (2013). The non-coding transcriptome of the hyperthermophilic archaeon Pyrococcus abyssi RNA at 92 ° C. RNA Biol.

[32] Schut GJ, Zhou J, Adams MWW (2001). DNA microarray analysis of the hyperthermophilic archaeon Pyrococcus furiosus: evidence for a new type of sulfur-reducing enzyme complex. J Bacteriol.

[33] Schut GJ, Bridger SL, Adams MWW (2007). Insights into the metabolism of elemental sulfur by the hyperthermophilic archaeon Pyrococcus furiosus: characterization of a coenzyme A- dependent NAD(P)H sulfur oxidoreductase. J Bacteriol.

[34] Santangelo TJ, Cuboňová L, Reeve JN (2011). Deletion of alternative pathways for reductant recycling in Thermococcus kodakarensis increases hydrogen production. Mol Microbiol.

[35] Kanai T, Matsuoka R, Beppu H, Nakajima A, Okada Y, Atomi H, Imanaka T (2011). Distinct physiological roles of the three [NiFe]-hydrogenase orthologs in the hyperthermophilic archaeon *Thermococcus kodakarensis*. J. Bacteriol.

[36] Hoffmann S, Otto C, Kurtz S, Sharma CM, Khaitovich P, Vogel J, Stadler PF, Hackermüller J (2009). Fast mapping of short sequences with mismatches, insertions and deletions using index structures. PLoS Comput Biol.

[37] Fukui T, Atomi H, Kanai T, Matsumi R, Fujiwara S, Imanaka T (2005). Complete genome sequence of the hyperthermophilic archaeon Thermococcus kodakarensis KOD1 and comparison with Pyrococcus genomes. Genome Res.

[38] Burge SW, Daub J, Eberhardt R, Tate J, Barquist L, Nawrocki EP, Eddy SR, Gardner PP, Bateman A (2013). Rfam 11.0: 10 years of RNA families. Nucl Acids Res.

[39] Chan PP, Holmes AD, Smith AM, Tran D, Lowe TM (2012). The UCSC archaeal genome browser: 2012 update. Nucl Acids Res.

[40] Mao F, Dam P, Chou J, Olman V, Xu Y (2009). DOOR: a database for prokaryotic operons. Nucl Acids Res.

[41] Mao X, Ma Q, Zhou C, Chen X, Zhang H, Yang J, Mao F, Lai W, Xu Y (2014). DOOR 2.0: presenting operons and their functions through dynamic and integrated views. Nucl Acids Res.

[42] Jeon SJ, Fujiwara S, Takagi M, Imanaka T (1999). Pk-cdcA encodes a CDC48/VCP homolog in the hyperthermophilic archaeon Pyrococcus kodakarensis KOD1: transcriptional and enzymatic characterization. Mol Gen Genet.

[43] Fujiwara S, Aki R, Yoshida M (2008). Expression profiles and physiological roles of two types of molecular chaperonins from the hyperthermophilic archaeon Thermococcus kodakarensis. Appl Env Microbiol.

[44] Shimada Y, Fukuda W, Akada Y, Ishida M, Nakayama J, Imanaka T, Fujiwara S (2009). Property of cold inducible DEAD-box RNA helicase in hyperthermophilic archaea. Biochem Biophys Res Commun.

[45] Bell SD, Jackson SP (2001). Mechanism and regulation of transcription in archaea. Curr Opin Microbiol.

[46] Bailey TL, Boden M, Buske FA, Frith M, Grant CE, Clementi L, Ren J, Li WW, Noble WS (2009). MEME SUITE: tools for motif discovery and searching. Nucl Acids Res.

[47] Brenneis M, Hering O, Lange C, Soppa J (2007). Experimental characterization of cis-acting elements important for translation and transcription in halophilic archaea. PLoS Genet.

[48] Hering O, Brenneis M, Beer J, Suess B, Soppa J (2009). A novel mechanism for translation initiation operates in haloarchaea. Mol Microbiol.

[49] La Teana A, Benelli D, Londei P, Bläsi U (2013). Translation initiation in the crenarchaeon Sulfolobus solfataricus: eukaryotic features but bacterial route. Biochem Soc Trans.

[50] Brenneis M, Soppa J (2009). Regulation of translation in haloarchaea: 5′- and 3′-UTRs are essential and have to functionally interact in vivo. PLoS One.

[51] Rodionov DA, Vitreschak AG, Mironov AA, Gelfand MS (2002). Comparative genomics of thiamin biosynthesis in procaryotes: new genes and regulatory mechanisms. J Biol Chem.

[52] Weinberg Z, Wang JX, Bogue J, Yang J, Corbino K, Moy RH, Breaker RR (2010). Comparative genomics reveals 104 candidate structured RNAs from bacteria, archaea, and their metagenomes. Genome Biol.

[53] Li S, Smith KD, Davis JH, Gordon PB, Breaker RR, Strobel SA (2013). Eukaryotic resistance to fluoride toxicity mediated by a widespread family of fluoride export proteins. Proc Natl Acad Sci USA.

[54] Loh E, Dussurget O, Gripenland J, Vaitkevicius K, Tiensuu T, Mandin P, Repoila F, Buchrieser C, Cossart P, Johansson J (2009). A trans-acting riboswitch controls expression of the virulence regulator PrfA in Listeria monocytogenes. Cell.

[55] Mellin JR, Tiensuu T, Bécavin C, Gouin E, Johansson J (2013). A riboswitch-regulated antisense RNA in Listeria monocytogenes. Proc Natl Acad Sci USA.

[56] Klein RJ, Misulovin Z, Eddy SR (2002). Noncoding RNA genes identified in AT-rich hyperthermophiles. Proc Natl Acad Sci USA.

[57] Pan M, Santangelo TJ, Li Z, Reeve JN, Kelman Z (2011). Thermococcus kodakarensis encodes three MCM homologs but only one is essential. Nucl Acids Res.

[58] Marchler-Bauer A, Zheng C, Chitsaz F, Derbyshire MK, Geer LY, Geer RC, Gonzales NR, Gwadz M, Hurwitz DI, Lanczycki CJ, Lu F, Lu S, Marchler GH, Song JS, Thanki N, Yamashita RA, Zhang D, Bryant SH (2013). CDD: conserved domains and protein three-dimensional structure. Nucl Acids Res.

[59] Georg J, Hess WR (2011). cis-antisense RNA, another level of gene regulation in bacteria. Microbiol Mol Biol Rev.

[60] Yoon SH, Reiss DJ, Bare JC, Tenenbaum D, Pan M, Slagel J, Moritz RL, Lim S, Hackett M, Menon AL, Adams MWW, Barnebey A, Yannone SM, Leigh J a, Baliga NS (2011). Parallel evolution of transcriptome architecture during genome reorganization. Genome Res.

[61] Tang TH, Bachellerie JP, Rozhdestvensky T, Bortolin ML, Huber H, Drungowski M, Elge T, Brosius J, Hüttenhofer A (2002). Identification of 86 candidates for small non-messenger RNAs from the archaeon Archaeoglobus fulgidus. Proc Natl Acad Sci USA.

[62] Tang TH, Polacek N, Zywicki M, Huber H, Brugger K, Garrett R, Bachellerie JPJP, Hüttenhofer A, Huttenhofer A (2005). Identification of novel non-coding RNAs as potential antisense regulators in the archaeon Sulfolobus solfataricus. Mol Microbiol.

[63] Straub J, Brenneis M, Jellen-Ritter A, Heyer R, Soppa J, Marchfelder A (2009). Small RNAs in haloarchaea: identification, differential expression and biological function. RNA Biol.

[64] Kanehisa M, Goto S (2000). KEGG: Kyoto encyclopedia of genes and genomes. Nucl Acids Res.

[65] Aoki-Kinoshita KF, Kanehisa M (2007). Gene annotation and pathway mapping in KEGG. Methods Mol Biol.

[66] Jäger D, Pernitzsch SR, Richter AS, Backofen R, Sharma CM, Schmitz RA (2012). An archaeal sRNA targeting cis- and trans-encoded mRNAs via two distinct domains. Nucleic Acids Res.

[67] Richter H, Mohr S, Randau L (2013). C/D box sRNA, CRISPR RNA and tRNA processing in an archaeon with a minimal fragmented genome. Biochem Soc Trans.

[68] Barbezier N, Canino G, Rodor J, Jobet E, Saez-Vasquez J, Marchfelder A, Echeverría M (2009). Processing of a dicistronic tRNA-snoRNA precursor: combined analysis in vitro and in vivo reveals alternate pathways and coupling to assembly of snoRNP. Plant Physiol.

[69] Baird NJ, Ferré-D’Amaré AR (2013). Modulation of quaternary structure and enhancement of ligand binding by the K-turn of tandem glycine riboswitches. RNA.

[70] Blouin S, Lafontaine DA (2007). A loop loop interaction and a K-turn motif located in the lysine aptamer domain are important for the riboswitch gene regulation control. RNA.

[71] Cho IM, Lai LB, Susanti D, Mukhopadhyay B, Gopalan V (2010). Ribosomal protein L7Ae is a subunit of archaeal RNase P. Proc Natl Acad Sci USA.

[72] Elmore JR, Yokooji Y, Sato T, Olson S, Glover CVC, Graveley BR, Atomi H, Terns RM, Terns MP (2013). Programmable plasmid interference by the CRISPR-Cas system in Thermococcus kodakarensis. RNA Biol.

[73] Schattner P (2002). Searching for RNA genes using base-composition statistics. Nucl Acids Res.

[74] Fischer S, Benz J, Späth B, Maier L-K, Straub J, Granzow M, Raabe M, Urlaub H, Hoffmann J, Brutschy B, Allers T, Soppa J, Marchfelder A (2010). The archaeal Lsm protein binds to small RNAs. J Biol Chem.

[75] Lipscomb GL, Keese AM, Cowart DM, Schut GJ, Thomm M, Adams MW, Scott RA (2009). SurR: a transcriptional activator and repressor controlling hydrogen and elemental sulphur metabolism in Pyrococcus furiosus. Mol Microbiol.

[76] Koide T, Reiss DJ, Bare JC, Pang WL, Facciotti MT, Schmid AK, Pan M, Marzolf B, Van PT, Lo F-Y, Pratap A, Deutsch EW, Peterson A, Martin D, Baliga NS (2009). Prevalence of transcription promoters within archaeal operons and coding sequences. Mol Syst Biol.

[77] Schubert S, Grünweller A, Erdmann VA, Kurreck J (2005). Local RNA target structure influences siRNA efficacy: systematic analysis of intentionally designed binding regions. J Mol Biol.

[78] Johnsson P, Lipovich L, Grandér D, Morris KV (1840). Evolutionary conservation of long non-coding RNAs; sequence, structure, function. Biochim Biophys Acta.

[79] Santangelo TJ, Čuboňová L, James CL, Reeve JN (2007). TFB1 or TFB2 is sufficient for Thermococcus kodakarensis viability and for basal transcription in vitro. J Mol Biol.

[80] Berezikov E, Thuemmler F, van Laake LW, Kondova I, Bontrop R, Cuppen E, Plasterk RH (2006). Diversity of microRNAs in human and chimpanzee brain. Nat Genet.

[81] Barrett T, Wilhite SE, Ledoux P, Evangelista C, Kim IF, Tomashevsky M, Marshall KA, Phillippy KH, Sherman PM, Holko M, Yefanov A, Lee H, Zhang N, Robertson CL, Serova N, Davis S, Soboleva A (2013). NCBI GEO: archive for functional genomics data sets–update. Nucl Acids Res.

[82] Thorvaldsdóttir H, Robinson JT, Mesirov JP (2013). Integrative genomics viewer (IGV): high-performance genomics data visualization and exploration. Brief Bioinform.

[83] **FASTX_Toolkit** http://hannonlab.cshl.edu/fastx_toolkit

[84] Förstner KU, Vogel J, Sharma CM (2014). READemption-a tool for the computational analysis of deep-sequencing-based transcriptome data. BioRxiv.

[85] Wickham H (2011). ggplot2. Wiley Interdiscip Rev Comput Stat.

[86] Anders S, Huber W (2010). Differential expression analysis for sequence count data. Genome Biol.

[87] Nawrocki EP, Eddy SR (2013). Infernal 1.1: 100-fold faster RNA homology searches. Bioinformatics.

[88] Lowe TM, Eddy SR (1999). A computational screen for methylation guide snoRNAs in yeast. Science.

[89] Hertel J, Hofacker IL, Stadler PF (2008). SnoReport: computational identification of snoRNAs with unknown targets. Bioinformatics.

[90] Camacho C, Coulouris G, Avagyan V, Ma N, Papadopoulos J, Bealer K, Madden TL (2009). BLAST+: architecture and applications. BMC Bioinformatics.

[91] Lorenz R, Bernhart SH, Höner zu Siederdissen C, Tafer H, Flamm C, Stadler PF, Hofacker IL (2001). ViennaRNA package 2.0. AMB.

