# Supplementary material for: Primary transcriptome map of the hyperthermophilic archaeon Thermococcus kodakarensis
Source: BMC Genomics. 2014 Aug 16;15(1):684. doi: 10.1186/1471-2164-15-684 (PMC4247193; doi:10.1186/1471-2164-15-684)
Supplement: Supplementary file 5 — Additional file 5: Figure S1: Sequences upstream of sRK28 and TK1195. The sequences upstream of sRK28 and TK1195 and sRk28 are shown with the promoter elements (BRE; TATA-box), and the TSS (+1) documented by dRNA-seq, identified in bold text. The region encoding sRK28 is highlighted in red. The sequence encoding the 5′-UTR (grey) and the GTG translation initiating codon of TK1195 are identified. (PDF 23 KB) [file 12864_2014_6679_MOESM5_ESM.pdf]

BRE **TATA** **+1 sRK28** **BRE** **TATA** **+1 TK1195**

5'-TGGAATG**GGCAA**GC**CTTTTATT**TGGTCGCGCGATAACCCCAAGT**G**CCCG-N_{69}**-**A**AGTAA**GG**TTTATAAG**AGACTTGTAAAAGGTCTGACGAGGC**G**AGACCG**GTG-3'**
