# Supplementary material for: Primary transcriptome map of the hyperthermophilic archaeon Thermococcus kodakarensis
Source: BMC Genomics. 2014 Aug 16;15(1):684. doi: 10.1186/1471-2164-15-684 (PMC4247193; doi:10.1186/1471-2164-15-684)
Supplement: Supplementary file 6 — Additional file 6: Figure S2: Secondary structure predictions of TEX resistant regions of transcripts from near the 3′-termini of genes. The sequences shown, transcribed from regions near the 3′-termini of the indicated genes, survived TEX digestion and so were prevalent in the dRNA-seq libraries. The secondary structures shown, and predicted stabilities, were generated by RNAfold 91. (PDF 32 KB) [file 12864_2014_6679_MOESM6_ESM.pdf]

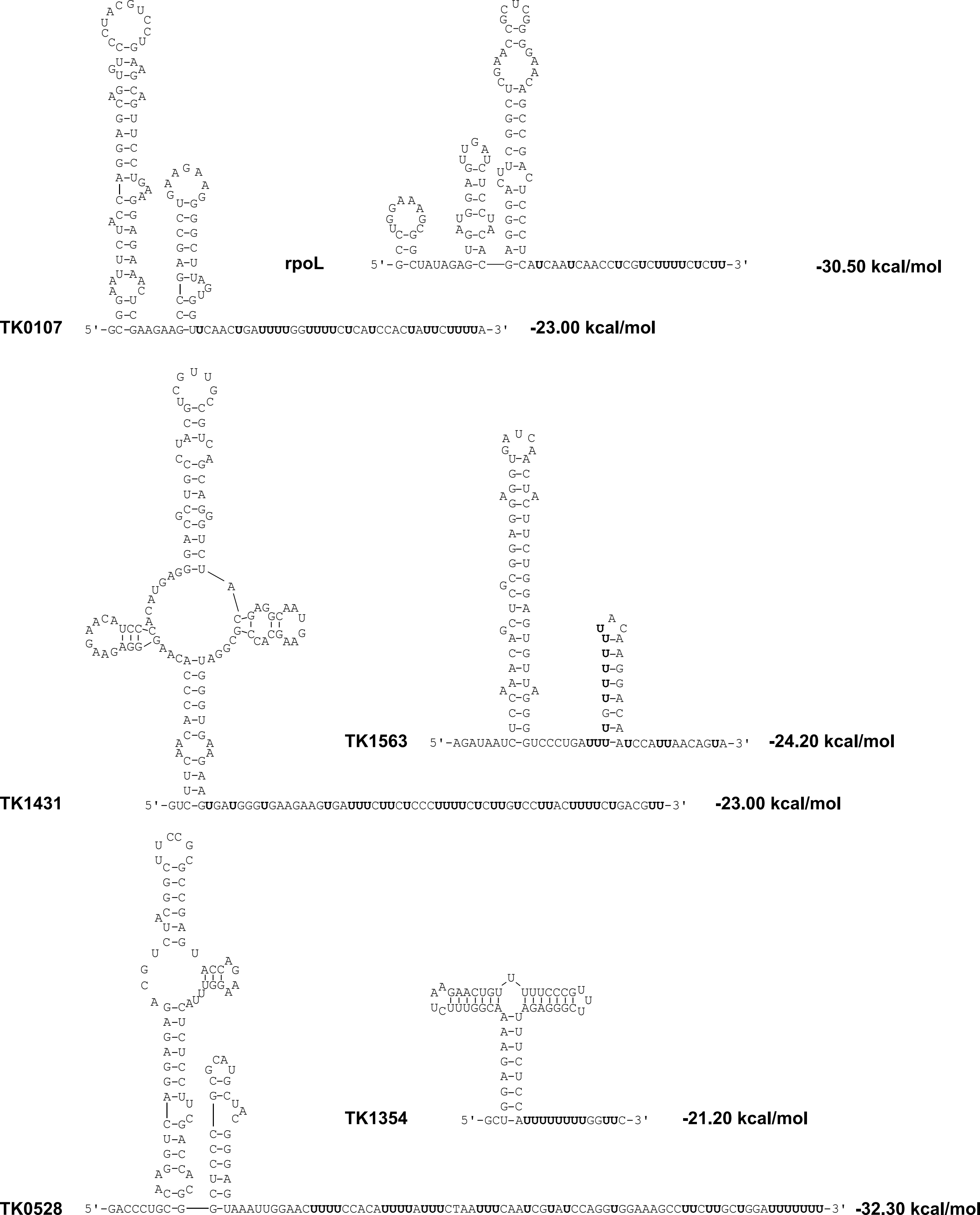

rpoL
5'-G-CUAUAGAG-C——G-CAUCAAUCAACCUCGUCUUUUCUCUU-3'
-30.50 kcal/mol
TK0107
5'-GC-GAAGAAG-UUCAACUGAUUUUGGUUUUCUCAUCCACUAUUCUUUUA-3'
-23.00 kcal/mol
TK1563
5'-AGAUAAUC-GUCCCUGAUUU-AUCCAUUAACAGUA-3'
-24.20 kcal/mol
TK1431
5'-GUC-GUGAUGGGUGAAGAAGUGAUUUCUUCUCCCUUUUCUCUUGUCCUUACUUUUCUGACGUU-3'
-23.00 kcal/mol
TK1354
5'-GCU-AUUUUUUUUGGUUC-3'
-21.20 kcal/mol
TK0528
5'-GACCCUGC-G——G-UAAAUUGGAACUUUUCCACAUUUUAUUUCUAAUUUCAAUCGUAUCCAGGUGGAAAGCCUUCUUGCUGGAUUUUUUU-3'
-32.30 kcal/mol
