# Supplementary figures and images for: Primary transcriptome map of the hyperthermophilic archaeon Thermococcus kodakarensis
Source: BMC Genomics. 2014 Aug 16;15(1):684. doi: 10.1186/1471-2164-15-684 (PMC4247193; doi:10.1186/1471-2164-15-684)

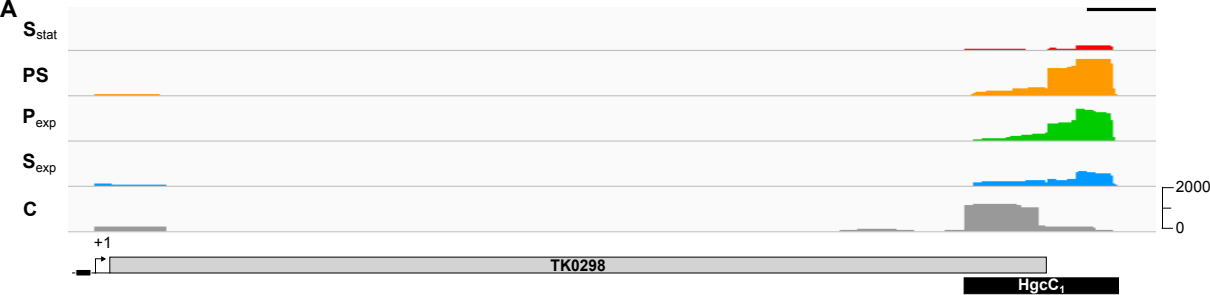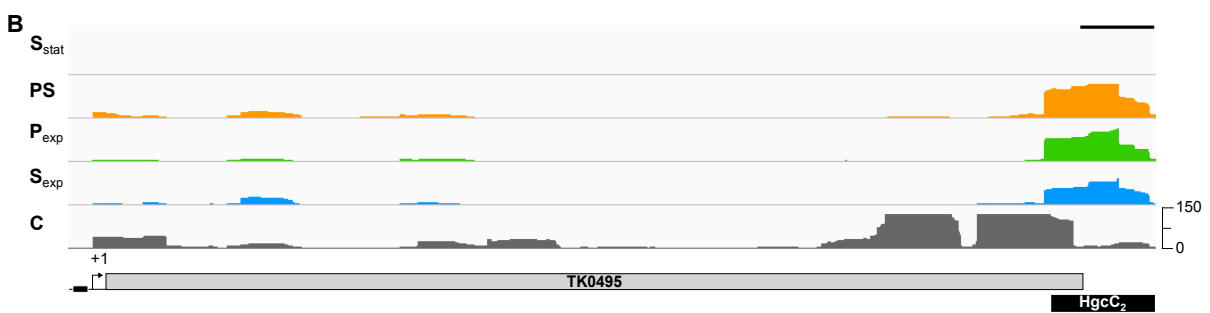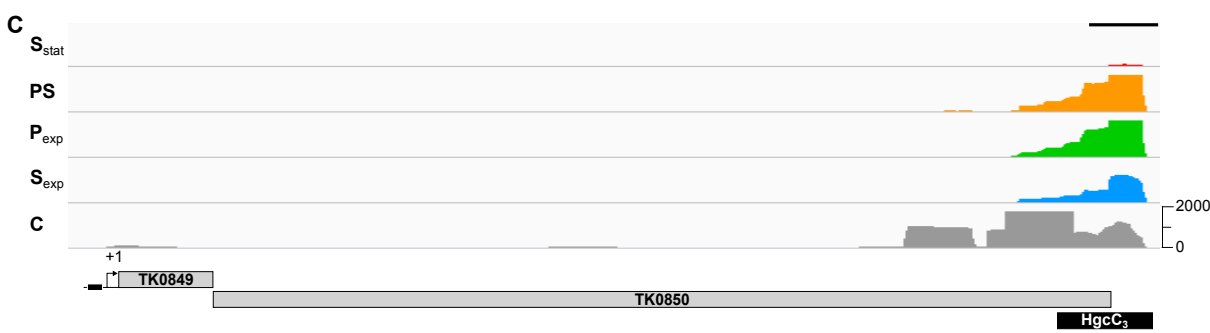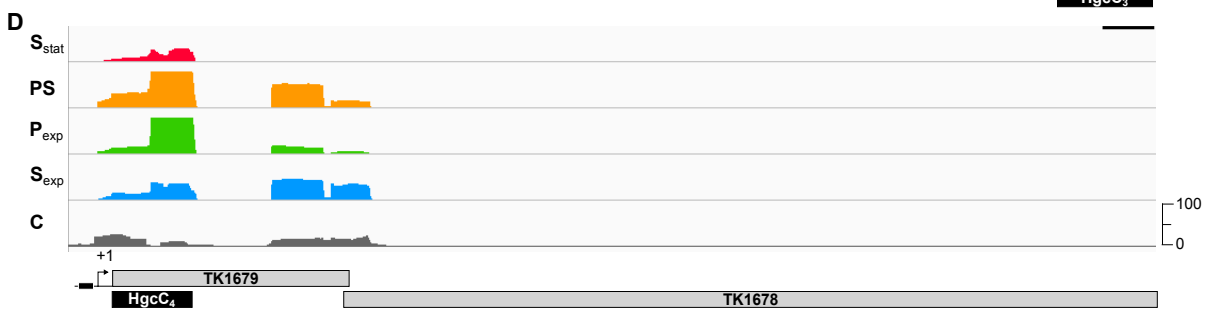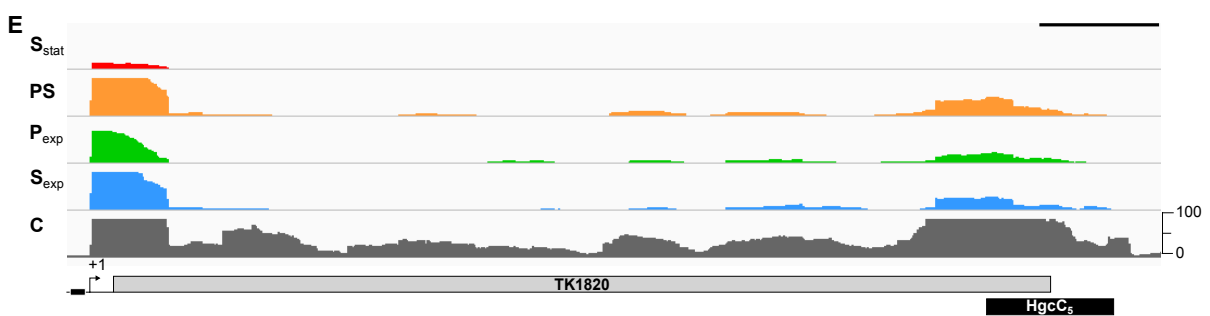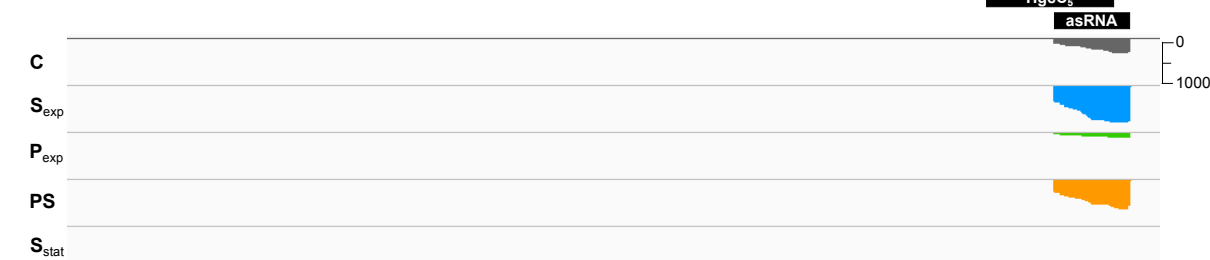

Supplement: Supplementary file 7 — Additional file 7: Figure S3: HgcC transcripts encoded in the T. kodakarensis genome. A-E. The genomic locations of five TK HgcC transcripts (black boxes). The abundances of transcripts synthesized from these regions in T. kodakarensis cells growing exponentially (Sexp; blue) and in stationary phase (Sstat; red) in sulfur medium, growing exponentially in pyruvate medium before (Pexp; green) and 20 min after sulfur addition (PS; orange) are given by the peak heights. Data from the control library (C) not digested with TEX are shown in grey. As illustrated, an antisense RNA is also transcribed from the region encoding HgcC5. The relative abundance scales on the right of each panel allow direct comparisons of all data in that panel. The black scale bar in the top right corner of each panel is corresponds to 100 nt. (PDF 1 MB) [file 12864_2014_6679_MOESM7_ESM.pdf]

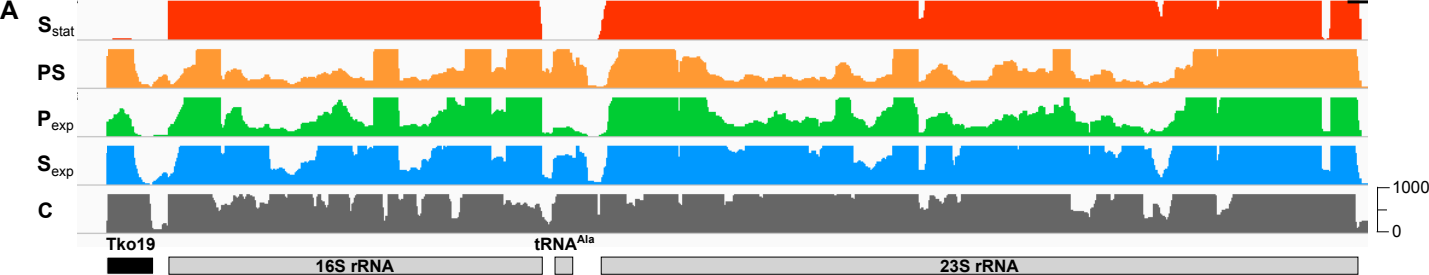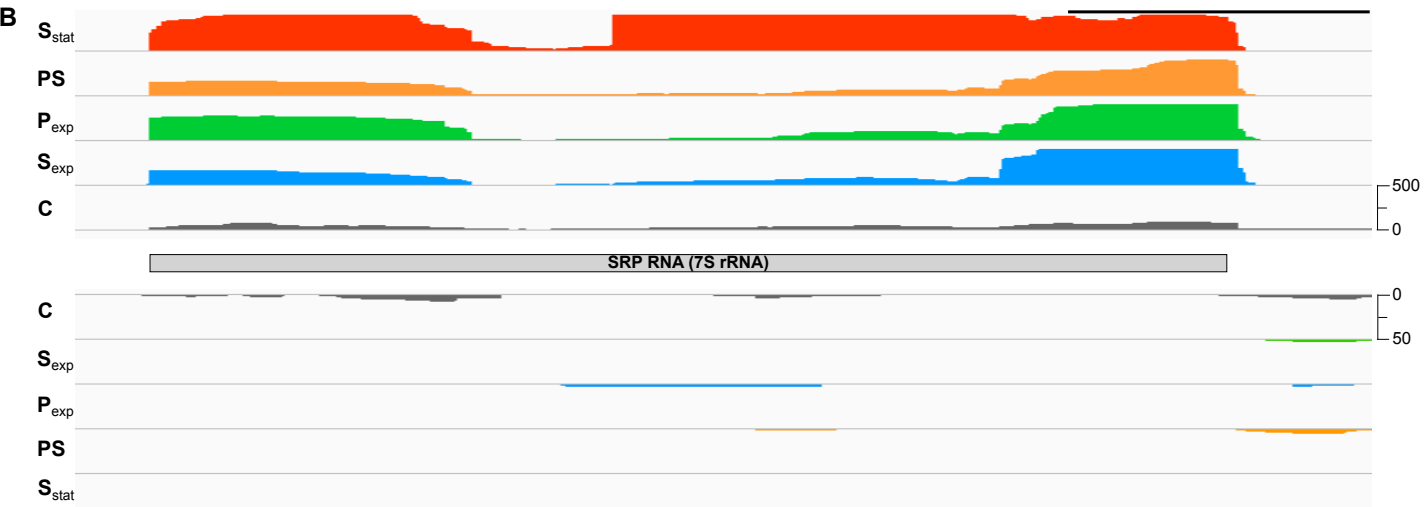

Supplement: Supplementary file 12 — Additional file 12: Figure S4: Transcription of the rRNA operon and SRP RNA. (A) As illustrated, the 16S-tRNAAla-23S rRNA operon is cotranscribed with a snoRNA, designated Tko19. (B) The SRP RNA is transcribed from the stand opposite that designated in the genome annotation [38]. The abundances of transcripts present in cells growing exponentially (Sexp; blue) and in stationary phase (Sstat; red) in sulfur medium, growing exponentially in pyruvate medium before (Pexp; green) and 20 min after sulfur addition (PS; orange) are given by the peak heights. Data from the control library (C) not digested with TEX are shown in grey. The relative abundance scales on the right of each panel allow direct comparisons of all data in that panel. The black scale bar in the top right corner of each panel is corresponds to 100 nt. (PDF 832 KB) [file 12864_2014_6679_MOESM12_ESM.pdf]

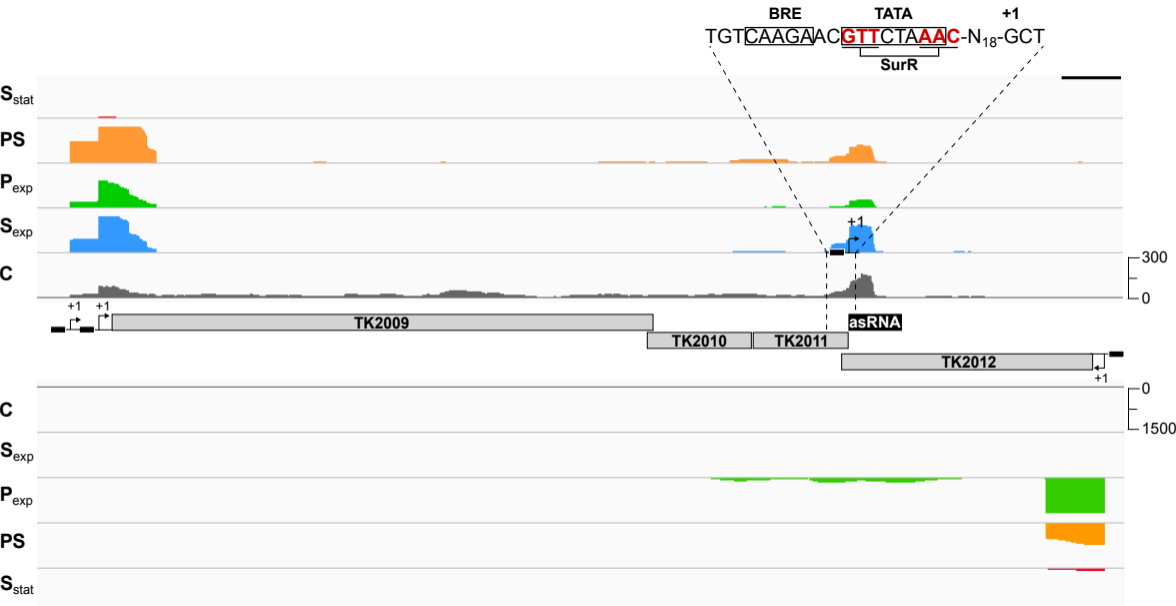

Supplement: Supplementary file 14 — Additional file 14: Figure S5: Genome organization around TK2012 (encodes ferredoxin-3). As illustrated, a putative SurR binding site (red font) overlaps the BRE-TATA-box region of a promoter that directs transcription of an antisense RNA (black box) from the TK2012 region. The abundances of transcripts present in cells growing exponentially (Sexp; blue) and in stationary phase (Sstat; red) in sulfur medium, growing exponentially in pyruvate medium before (Pexp; green) and 20 min after sulfur addition (PS; orange) are given by the peak heights. Data from the control library (C) not digested with TEX are shown in grey. The relative abundance scales on the right of each panel allow direct comparisons of all data in that panel. The black scale bar in the top right corner of each panel is corresponds to 100 nt. (PDF 309 KB) [file 12864_2014_6679_MOESM14_ESM.pdf]
